# Tip60 HAT Activity Mediates APP Induced Lethality and Apoptotic Cell Death in the CNS of a *Drosophila* Alzheimer's Disease Model

**DOI:** 10.1371/journal.pone.0041776

**Published:** 2012-07-26

**Authors:** Sheila K. Pirooznia, Jessica Sarthi, Ashley A. Johnson, Meridith S. Toth, Kellie Chiu, Sravanthi Koduri, Felice Elefant

**Affiliations:** Department of Biology, Drexel University, Philadelphia, Pennsylvania, United States of America; Oregon Health and Science University, United States of America

## Abstract

Histone acetylation of chromatin promotes dynamic transcriptional responses in neurons that influence neuroplasticity critical for cognitive ability. It has been demonstrated that Tip60 histone acetyltransferase (HAT) activity is involved in the transcriptional regulation of genes enriched for neuronal function as well as the control of synaptic plasticity. Accordingly, Tip60 has been implicated in the neurodegenerative disorder Alzheimer's disease (AD) *via* transcriptional regulatory complex formation with the AD linked amyloid precursor protein (APP) intracellular domain (AICD). As such, inappropriate complex formation may contribute to AD-linked neurodegeneration by misregulation of target genes involved in neurogenesis; however, a direct and causative epigenetic based role for Tip60 HAT activity in this process during neuronal development *in vivo* remains unclear. Here, we demonstrate that nervous system specific loss of Tip60 HAT activity enhances APP mediated lethality and neuronal apoptotic cell death in the central nervous system (CNS) of a transgenic AD fly model while remarkably, overexpression of Tip60 diminishes these defects. Notably, all of these effects are dependent upon the C-terminus of APP that is required for transcriptional regulatory complex formation with Tip60. Importantly, we show that the expression of certain AD linked Tip60 gene targets critical for regulating apoptotic pathways are modified in the presence of APP. Our results are the first to demonstrate a functional interaction between Tip60 and APP in mediating nervous system development and apoptotic neuronal cell death in the CNS of an AD fly model *in vivo*, and support a novel neuroprotective role for Tip60 HAT activity in AD neurodegenerative pathology.

## Introduction

Epigenetic regulation of chromatin structure *via* histone acetylation promotes coordinated and dynamic transcriptional responses in neurons that influence the neuroplasticity critical for cognitive ability [1]. Tip60 is a cellular acetyltransferase protein that was originally identified by its interaction with the HIV-1 transactivator protein Tat [2]. As such, a role for Tip60 in transcription regulation has been investigated intensively with accumulating data linking Tip60 to diverse processes including cell signaling, DNA damage repair, cell cycle and checkpoint control and apoptosis [3]. Recent work from our laboratory demonstrates that the HAT activity of Tip60 is required for the transcriptional regulation of genes enriched for neuronal function [4] as well as the regulation of synaptic plasticity [5]. Consistent with these findings, Tip60 has been implicated in the neurodegenerative disorder Alzheimer's disease (AD) *via* its formation of a transcriptional regulatory complex with the AD linked amyloid precursor protein (APP) intracellular domain (AICD). It has been demonstrated that this complex is recruited to the promoters of certain target genes where it acts to acetylate select histone proteins to epigenetically regulate gene transcription [6]–[9]. Importantly, aberrant expression of some of these genes has been linked to AD pathophysiology [10]–[12]. Based on these findings, it has been proposed that inappropriate complex formation and/or recruitment may contribute or lead to AD pathology *via* misregulation of target genes required for neurogenesis. Growing evidence suggests that the cognitive impairment in AD as well as signaling between neurons is interrupted at early stages of the disease [13]. It has also been hypothesized that dysregulation of epigenetic control mechanisms and the resultant aberrant epigenetic marks may contribute to such cognitive dysfunction [14]. However, a direct and causative epigenetic based role for Tip60 HAT activity misregulation in disrupting APP mediated neuronal processes linked to AD during nervous system development *in vivo* remains to be tested.

Apoptosis or programmed cell death is crucial in guiding the physiological development of individual cells and organs and is particularly important for CNS development [15]. Misregulation of this process leads to inappropriate induction of neuronal specific apoptotic cell death that has been shown to be a hallmark of certain progressive neurodegenerative diseases, one of which is AD. Importantly, Tip60 and AICD have each been shown to play separate and critical roles in the induction of apoptosis. For example, Tip60 plays a central role as a primary cell cycle mediator by modulating the direction of p53-dependent cell fate towards either cell cycle arrest or apoptotic induction. Tip60 carries out this role by first sensing the level of irrepairable DNA damage, and then inducing the appropriate p53-dependant response pathway via its HAT activity [16]. Interestingly, the Tip60 interacting γ-secretase derived APP intracellular C-terminal domain (AICD) fragment has also been shown to trigger p53-dependent cell death by increasing p53 expression and activity in human brain and neuronal cell models [17]. Additionally, ectopic expression of AICD in H4 neuroglioma cells leads to dramatic nuclear localization and apoptosis [18]. Moreover, mutations in the presenilin proteins of the AICD generating γ-secretase complex are also linked to neurodegeneration and AD progression [19]–[22]. However, despite the convincing evidence that Tip60 and APP are each separately involved in promoting neuronal apoptotic induction, a functional interaction between Tip60 and APP in the control of this process remains to be explored, and an *in vivo* model to test this hypothesis has yet to be generated.

In this report, we test the hypothesis that Tip60 HAT activity mediates APP induced lethality and apoptotic neuronal cell death in the central nervous system (CNS) using a transgenic AD fly model that we uniquely adapted to express varying levels of Tip60 HAT activity. We demonstrate that nervous system specific loss of Tip60 HAT activity enhances APP mediated lethality and neuronal apoptotic cell death in the developing central nervous system (CNS) of these transgenic flies while remarkably, overexpression of Tip60 counteracts these defects. Notably, all of these effects are dependent upon the APP C-terminal domain that is required for transcriptional regulatory complex formation with Tip60. Importantly, we show that the expression of certain AD linked Tip60 gene targets critical for regulating apoptotic pathways are modified in the presence of APP. Our findings are the first to show a functional interaction between Tip60 HAT activity and APP in mediating both nervous system development and apoptosis linked neuronal cell death in the CNS of an AD fly model *in vivo*, and point to a novel neuroprotective role for Tip60 HAT activity in AD neurodegenerative pathology.

## Materials and Methods

### *Drosophila* Genetics

*Drosophila* stocks were maintained at 25°C on standard cornmeal/agar/molasses medium supplemented with yeast. The *w^1118^* line served as the genetic background control. The generation and characterization of the dominant negative HAT mutant dTIP60^E431Q^ lines A and B is described in [4]. Transgenic UAS lines carrying human APP 695 isoform (UAS-APP) and APP lacking the C-terminus (UAS-APP dCT) were obtained from *Drosophila* Stock Center (Bloomington, IN, USA). Stocks carrying dTIP60^E431Q^ lines A or B were introduced into UAS-APP and UAS-APP dCT backgrounds using standard genetic techniques. As previously described [4], transgenic UAS fly lines that would allow for expression of varying levels of wild type *Drosophila* Tip60 (dTip60^WT^) were generated and crossed into both UAS-APP and UAS-APP dCT backgrounds using standard genetic techniques. The ubiquitously expressed 337-Gal4 driver and the nervous system specific 179 y-Gal4 driver were obtained from *Drosophila* Stock Center (Bloomington, IN, USA). Viability analysis was performed using newly eclosed age matched virgin females. For ubiquitous expression of the different transgenic lines, ten virgin females from each of the lines were crossed to seven 337-Gal4 males. The crosses were maintained at 25°C and transferred to fresh food every 24 hrs for 3 days. Each transfer was counted as day 1. The crosses were monitored daily and the developmental stage at which lethality (if any) occurred was recorded. The number of flies that eclosed were counted daily starting on day 10 for a period of ten days at which point all the F1 progeny had either eclosed or died as pupae. The average number of flies for the three days was calculated. For each transgenic line, three replicate crosses were done as described above and the developmental stage at which lethality occurs as well as average number of eclosed flies were reported. The same was repeated for nervous system specific expression of the different transgenes using ten newly eclosed age matched 179 y-Gal4 females and seven males from each of the transgenic lines.

### Quantitative Real Time RT-PCR

Quantification of RNA transcript levels of dTip60^E431Q^ or dTip60^WT^ in the different double transgenic lines was done by crossing the respective fly lines to 337-Gal4 driver at 25°C as described earlier. As a control, W^1118^ flies were crossed to 337-Gal4 flies. Staged F1 second instar larvae that resulted from the cross were used for RNA extraction. Total RNA was isolated using Trizol (Invitrogen Corporation, Carlsbad, CA, USA) and treated twice with DNase II (Ambion, Austin, TX) to remove DNA. Complementary DNA (cDNA) was synthesized from 1 ug total RNA and oligo-dT primers using Superscript II Reverse Transcriptase (Invitrogen Corporation, Carlsbad, CA,USA). Real-time quantitative PCR was performed on an ABI 7500 Real Time PCR System (Applied Biosystems, Poster City, CA, USA) using the Power SYBR Green PCR master mix (Applied Bioystems, Poster City, CA, USA). Real time RT-PCR reactions were carried out in triplicate in 20 ul reaction volumes containing 1 ng cDNA template and 1.5 uM each of forward and reverse primer. Transgene induced expression of exogenous dTip60^E431Q^ or dTip60^WT^ for each line was determined as described in Lorbeck *et al* (2010) by amplifying total dTip60 mRNA using primers designed to amplify a non-conserved region within both the endogenous dTip60 and exogenous transgene induced dTip60, and comparing the relative fold change in mRNA expression levels to just the endogenous dTip60 mRNA level that was determined using primers that amplify the endogenous 5′UTR dTip60 region that is lacking in the exogenously expressed dTip60. Forward and reverse primer sets designed to amplify a 97 bp nonconserved region of dTIP60 were 5′GACGGCTCACAAACAGGC 3′and 5′GGTGTTGCGGTGATGTAGG 3′, respectively. Forward and reverse primers designed to amplify a 105 bp region within the 5′UTR region of endogenous dTIP60 were 5′CAGTTGTGGTT CACAATTACCC 3′ and 5′GTGCGCAGAAAGTTATACAGC 3′, respectively. PCR was carried out by 40 cycles at 95°C for 45 sec, 55°C for 45 sec, and 72°C for 1 min with plate readings recorded after each cycle. Threshold cycle (Ct) values were obtained, and the ΔΔCT method [23] was used to calculate the fold change in transcript level of the sample relative to the control. RP49 which encodes the *Drosophila* ribosomal protein L32 was used as an internal standard and reference gene using forward and reverse primer pairs 5′CTGCTCATGCAGAACCGCGT 3′and 5′GGACCGACAGCTGCTTGGCG 3′, respectively.

### Semi-quantitative RT-PCR analysis

The presence of UAS-APP or UAS-APP dCT constructs in the double transgenic lines was verified by semi-quantitative RT-PCR. Total RNA and cDNA preparation from staged second instar larvae was done as before. PCR amplification was done in 20 ul reactions using forward and reverse primer pairs 5′-GCCGTGGCATTCTTTTGGGGC-3′ and 5′- GTGGTCAGTCCTCGGTCGGC-3′, respectively that amplify a 100 bp region in the APP N-terminus region. The PCR reaction mixture contained reaction buffer (10 mM Tris-HCl [pH 9.0], 50 mM KCl, 3 mM MgCl2 and 0.01% Triton X-100), 200 uM dNTPs, 1.5 uM of each primer, 1.25 U DNA polymerase (Qiagen, Hilden), and cDNA template. Thermal cycling conditions consisted of an initial melting step at 95°C for 1 min, followed by 39 cycles of melting at 95°C for 45 s, annealing at 55°C for 45 s and extension at 72°C for 60 s. PCR products were visualized by agarose gel (2%) electrophoresis containing ethidium bromide.

### TUNEL Staining for Apoptosis

Third instar larval brains were carefully dissected and fixed in 4% Paraformaldehyde. Brains were washed 3 times in 1× PBST (0.1% Triton X) for 15 minutes and incubated for 15 minutes in block solution (5% normal goat serum, 0.1% Triton X). Detection of apoptotic neuronal cells was performed using the Fluorescein Cell Death Kit (Roche, Mannheim, Germany) following the manufacturer's instructions. The reaction mixture was made using enzyme solution and label solution (1∶9) and brains were incubated for 90 minutes at 37°C. Samples were then washed three times in 1× PBST and mounted in Vectashield anti-fade mounting medium. Confocal microscopy was performed using Olympus Microscope with fluoview software. For each genotype including the wild type control, the replicate samples were dissected, fixed and stained on the same day using aliquots of enzyme reaction mixtures prepared from the same buffer/enzyme stock. The samples were protected from light and were also imaged within 24 hrs of preparing the slides to avoid loss of signal. Confocal imaging of whole-CNS was done by maintaining PMT voltage, offset, and laser power settings the same for the replicate samples in each case. Larval brain images were displayed as projections of 1 uM serial Z sections and represent whole compressed Z-stacks of the larval central nervous system.

### Microarray experiment

The experimental condition that was compared in the microarray experiment was wild type (WT) versus dTip60 ^E431Q^ B. As described previously (Lorbeck et al., 2011), respective flies were crossed to 337-Gal4 driver to allow for ubiquitous expression of the transgene. In each case, two samples of thirty-five staged three day old whole larvae progeny were used for RNA extraction and probing two separate microarray chips on the GeneChip Drosophila 2.0 Array (Affymetrix, Santa Clara, CA) following a standard Affymetrix protocol.

### Microarray data analysis

GeneChip CEL files were generated using the Affymetrix GeneChip operating system (GCOS). The CEL files are available at NCBI GEO (GEO Acc num. GSE25635). The open source packages in R and bioconductor were used for data analysis. The data were imported into R and after a series of pre-processing analysis (background correction and mean scaling), the data was normalized. The RMA normalization [24] which has been shown to have high efficiency for Affymetrix data normalization was chosen to minimize the systematic variation in the experiment. Limma (Linear Models for Microarray Data) package was used for detection of differentially expressed genes by fitting a linear model to the expression data for each gene. This package fully models the systematic part of the data and creates a design matrix. Each row of the design matrix corresponds to an array in the experiment and each column corresponds to a coefficient. In Affymetrix analysis, the linear modeling implemented by Limma is much the same as ordinary ANOVA or multiple regression except that a model is fitted for every gene. A list of the top genes which show evidence of differential expression between the dTip60 ^E431Q^ B and WT was then generated by estimating the fold change of dTip60 ^E431Q^ B over WT. The results of the linear model were then summarized, and the p-values for multiple testing adjusted using a FDR (Benjamini and Hochberg's method) threshold of 0.05. The genes whose *P-*value of the log ratio are over 95% were categorized as ‘no-change’ in gene expression and the genes with expression levels that have a significant difference between the dTip60 ^E431Q^ B and WT (*P*<0.05) are either ‘up or down-regulated’. Thus genes which have positive log ratios of dTip60 ^E431Q^ B/WT are up-regulated in dTip60 ^E431Q^ B while genes with negative log ratios are down-regulated in dTip60 ^E431Q^ B. The misregulated genes were analyzed using Gene Ontology (www.geneontology.com) and the panther protein classification system (www.pantherdb.org) to identify apoptosis related genes that were significantly enriched in the microarray dataset.

### Quantitative RT-PCR analysis of microarray targets

Apoptosis related genes that were found to be significantly misregulated in response to loss of Tip60 HAT activity in the microarray analysis were further validated by quantitative RT-PCR in the following transgenic fly lines: dTip60^E431Q^, dTip60^WT^, APP; dTip60^E431Q^, APP; dTip60^WT^. In each case, F1 second instar larvae resulting from a cross between each of these transgenic fly line and 337-Gal4 driver were used for cDNA preparation. Wild type *w^1118^* flies crossed to 337-Gal4 driver were used as control. Primer sets were designed using NCBI/Primer-BLAST (www.ncbi.nlm.nih.gov/tools/primer-blast/). Primer sequences are available upon request. Fold change of the respective transcript level in the sample was calculated relative to the control by the ΔΔCT method using RP49 as internal control.

## Results

### Tip60 and APP functionally interact to mediate both general and nervous system specific development

**Figure 1:**
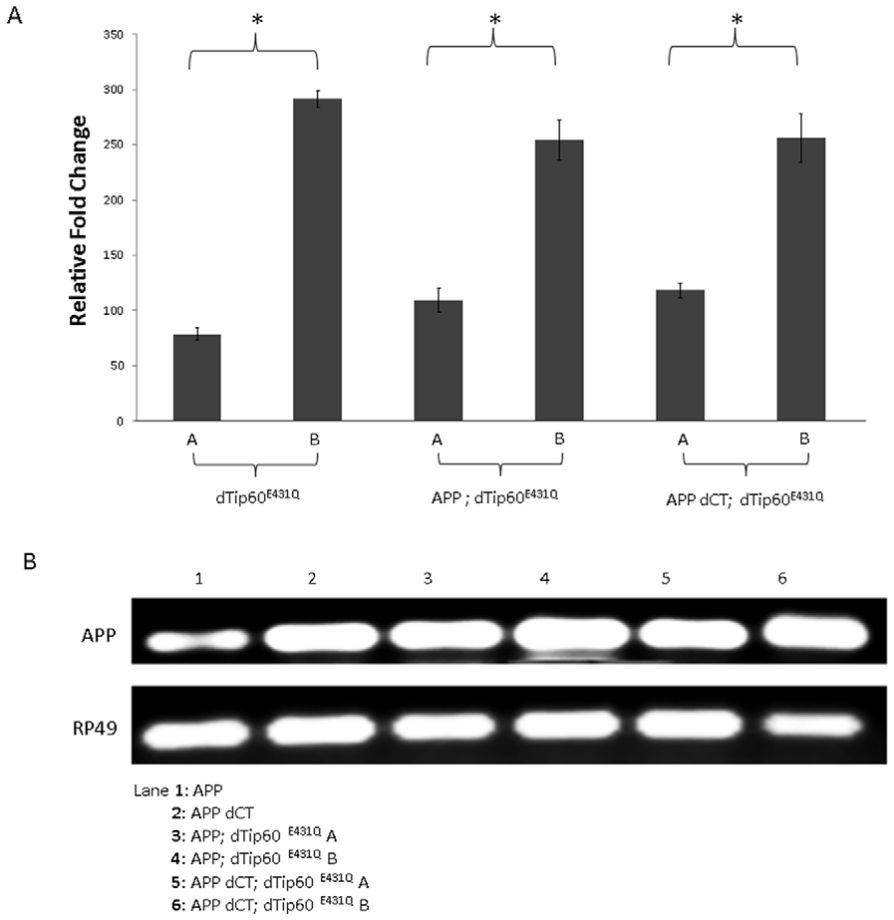
Generation and characterization of dTip60^E431Q^ containing APP or APP-dCT double transgenic flies. The dominant negative HAT defective lines dTip60^E431Q^ A or dTip60^E431Q^ B (Lorbeck *et al.*, 2011) were introduced into an APP or APP dCT background using standard genetic techniques. (**A**) Histogram depicting qPCR analysis of exogenous levels of dTip60^E431Q^ in staged F1 second instar larval progeny resulting from a cross between the ubiquitous driver 337 and either dTip60^E431Q^ (lines A and B), APP; dTip60^E431Q^ (lines A and B) or APP dCT; dTip60^E431Q^ (lines A and B). 337-Gal4 crossed to *w^1118^* served as a control. Quantification of the exogenously expressed dTip60^E431Q^ mRNA levels relative to endogenously expressed dTip60 mRNA was done using the comparative CT method with RP49 as internal control as described in (Lorbeck *et al*, 2011). Asterisks (*) indicate significant fold change between the lines A and B for each genotype with values of p<0.05; n = 3. Error bars represent standard error of the mean. (**B**) Semiquantitative RT-PCR analysis of APP or APP dCT expression in the different transgenic lines to confirm APP transgene presence. cDNA was prepared as before from staged second instar larvae ubiquitously expressing dTip60^E431Q^ with APP or APP dCT (lines A or B in each case) and PCR amplified using primers that flank a 100 bp region in the N-terminal portion of APP. PCR products were visualized using 2% agarose gel containing ethidium bromide. Staged second instar larvae ubiquitously expressing APP or APP dCT were used as controls.

**Figure 2:**
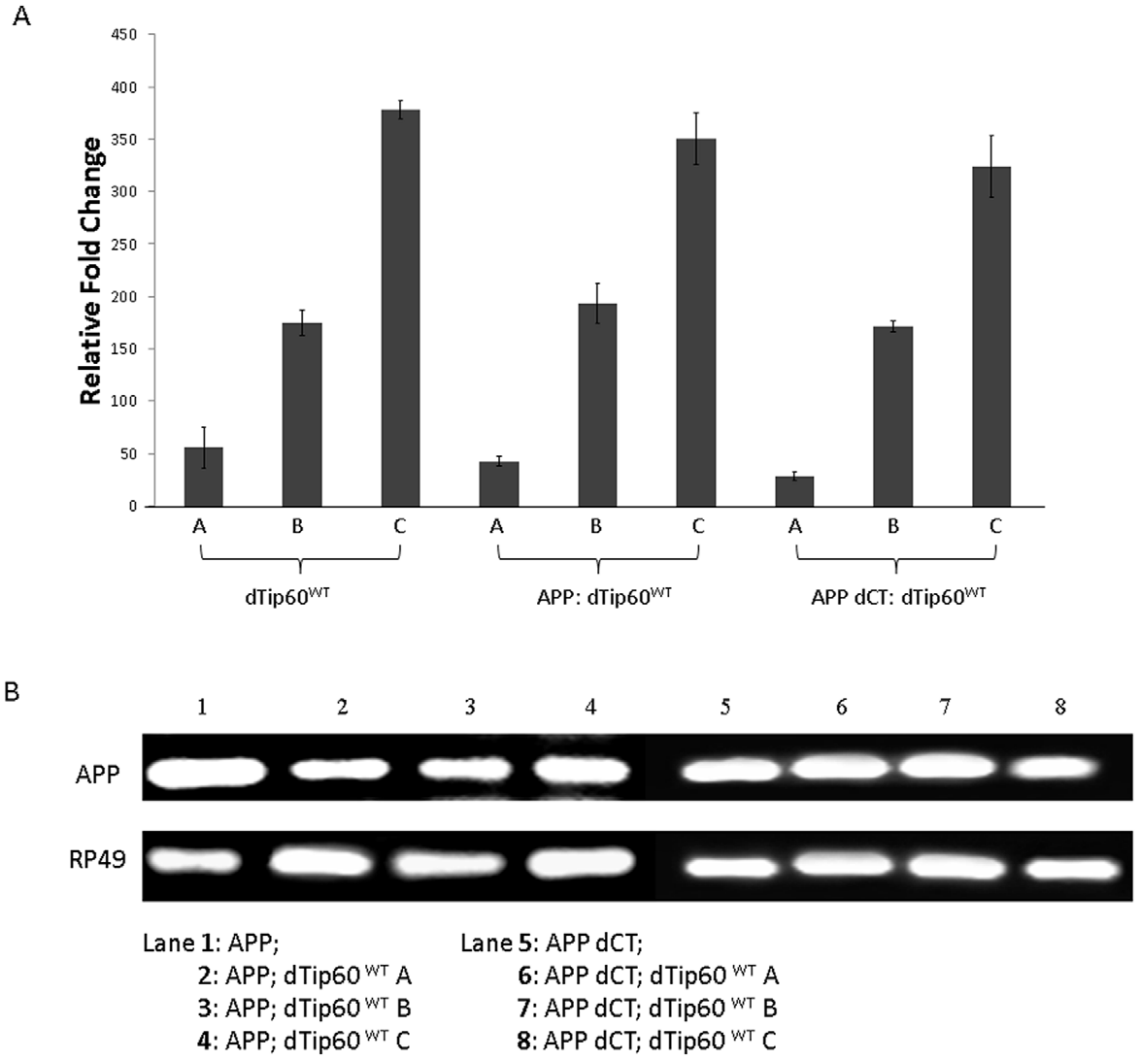
Generation and characterization of dTip60^WT^ containing APP or APP-dCT double transgenic flies. Flies expressing varying levels of wild type dTip60 (low, medium and high) were generated and then each introduced into APP or APP dCT background using standard genetic techniques. (**A**) The amount of wild type dTip60 that is exogenously induced relative to endogenous dTip60 was quantified by RT-PCR analysis of staged F1 second instar larvae resulting from the a cross between the ubiquitous driver 337 and either dTip60^WT^ (lines A, B and C), APP; dTip60^WT^ (lines A, B and C) or APP dCT; dTip60^WT^ (lines A, B and C). 337-Gal4 crossed to *w^1118^* was used as control. The relative fold change in mRNA expression levels between exogenous and endogenous dTip60 was measured as described before using the comparative CT method with RP49 as the internal control, and these results are summarized in the histogram. The amount of exogenously induced wild type dTip60 levels is significantly different between lines A, B and C in each case with values of p<0.05; n = 3. Error bars represent standard error of the mean. (**B**) Semi-quantitative RT-PCR analysis of APP or APP dCT expression in the different dTip60^WT^ containing transgenic lines to confirm APP transgene presence. cDNA was prepared as before from staged second instar larvae ubiquitously expressing dTip60^WT^ with APP or APP dCT (lines A, B or C in each case) and PCR amplified using primers that flank a 100 bp region in the N-terminal portion of APP. PCR products were visualized using 2% agarose gel containing ethidium bromide. Staged second instar larvae ubiquitously expressing APP or APP dCT were used as controls.

To create an *in vivo* multicellular model system suitable for investigating a functional link between Tip60 HAT activity and APP in neuronal function *in vivo*, we generated transgenic flies expressing either our previously characterized HAT-defective dominant negative Tip60 transgene (dTIP60^E431Q^) or additional copies of wild-type Tip60 transgene (dTip60^WT^) in a well characterized AD fly model [25], [26] that overexpresses either full-length human APP (APP) or human APP lacking the Tip60-interacting C-terminal domain (APP dCT) under the control of the UAS promoter. Double transgenic lines were generated for two independent dTip60^E431Q^ lines expressing low and high levels of the HAT activity defective mutant dTip60 (dTip60^E431Q^ A and dTip60^E431Q^ B, respectively) (Table 1). Similarly, double transgenic lines for three independent dTip60^WT^ lines expressing varying levels of wild type dTip60 (dTip60^WT^ A, dTip60^WT^ B, and dTip60^WT^ C, respectively) were generated (Table 1). Expression levels for the exogenously expressed dTip60^E431Q^ or dTip60^WT^ from each of these transgenic lines were quantitatively assessed using quantitative RT-PCR to allow for selection of lines that had comparable levels of exogenous Tip60^E431Q^ and Tip60^WT^ expression for further analysis (Figure 1A and Figure 2A). Comparable levels of APP and APP dCT transgene expression were previously characterized [26] and presence of each of these transgenes in the APP; dTip60 fly lines was confirmed using semi-quantitative PCR (Figure 1B and Figure 2B).

**Table 1:** Transgenic fly lines used for this study.

Transgenic fly lines^a^	Source^b^
UAS-dTip60^E431Q^ A	Lorbeck et al., 2011
UAS-APP; dTip60^E431Q^ A	This study
UAS-APP dCT: dTip60^E431Q^ A	
UAS-dTip60^E431Q^ B	Lorbeck et al., 2011
UAS-APP; dTip60^E431Q^ B	This study
UAS-APP dCT; dTip60^E431Q^ B	
UAS-dTip60^WT^ A	
UAS-dTip60^WT^ B	
UAS-dTip60^WT^ C	
UAS-APP; dTip60^WT^ A	
UAS-APP; dTip60^WT^ B	
UAS-APP; dTip60^WT^ C	
UAS-APP dCT; dTip60^WT^ A	
UAS-APP dCT; dTip60^WT^ B	
UAS-APP dCT; dTip60^WT^ C	

a The Tip60 P-element insertion is located on chromosome 3 and the APP P-element insertion is located on chromosome 2.

b Indicates where the transgenic fly lines were generated.

**Figure 3:**
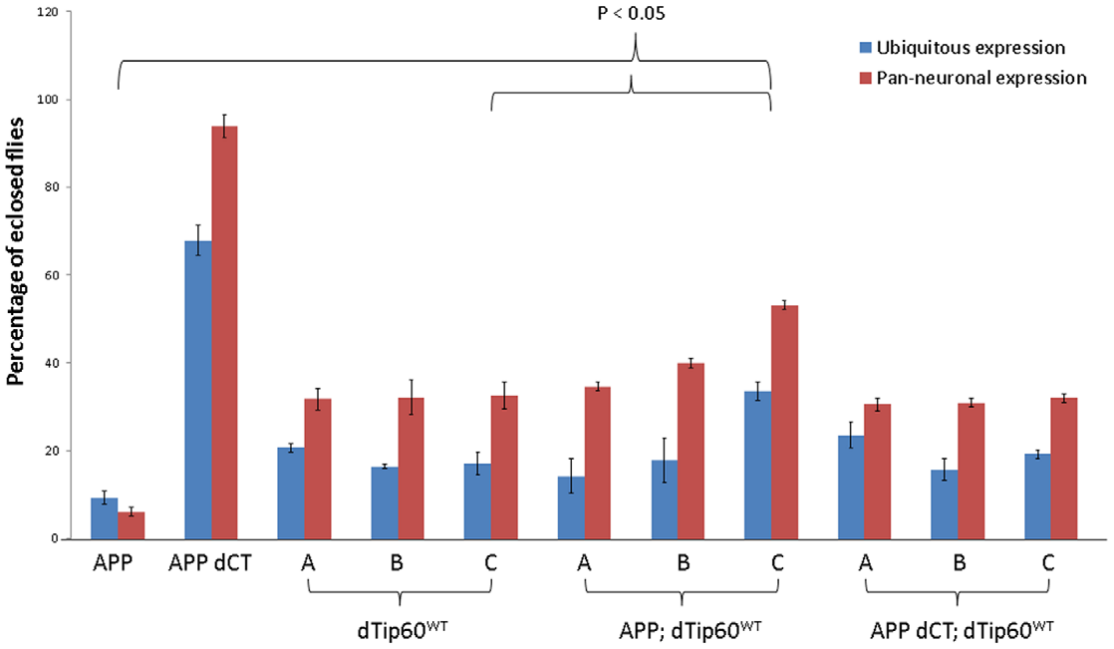
Viability analysis indicates genetic interaction between Tip60 and APP in *Drosophila*. The indicated transgene was expressed ubiquitously in the fly using 337-Gal4 driver or pan-neuronally using 179 y-Gal4 driver. The number of F1 progeny that eclosed were counted daily. The percentage of eclosed flies was calculated relative to the wild type control (*w^1118^*). All crosses were carried out in triplicate at 25°C. Overexpression of APP drastically reduced viability to <10% while no effect was observed due to expression of truncated version of APP lacking its C-terminal domain. Overexpression of varying levels of wild type dTip60 (dTip60^WT^) also reduced viability in a dose independent manner. However, co-expression of dTip60^WT^ with APP partially rescued the lethal effects induced by APP expression in a dose dependent manner with the maximum effect observed with high levels of dTip60^WT^. In the presence of APP lacking the C-terminus, overexpression of dTip60^WT^ had similar effects seen in flies that overexpressed dTip60^WT^ alone.

To determine whether Tip60 and APP functionally interact during general *Drosophila* development, we first expressed each of the transgenes (dTip60^E431Q^, dTip60^WT^, APP, APP-dCT) separately at the normal physiological temperature of 25°C using GAL4 driver line 337, that induces robust and ubiquitous GAL4 expression beginning during late embryogenesis and continuing into adulthood. The crosses were monitored daily to examine if the expression of the different transgenes affects development. In cases where the transgene expression induced lethality, the developmental stage at which lethality occurred was recorded (Table 2). In cases where the F1 progeny progressed through normal development and eclosed, the number of flies that eclosed over a ten day period were counted (Figure 3). The *w^1118^* fly line crossed to 337-GAL4 served as a control. As we previously reported, induction of Tip60^E431Q^ for both independent lines A and B reduced fly viability to 0%, with the majority of lethality occurring during the late third instar larval stage. Moreover, ubiquitous induction of APP resulted in 60% lethality that occurred in the pupal stage, with the remaining 40% of progeny surviving only 2–5 days after eclosion (Table 2, Figure 3). Co-expression of dTip60^E431Q^ and APP using both APP; dTip60^E431Q^ line A and APP; dTip60^E431Q^ line B resulted in 0% viability, with lethality occurring during the early second instar larval stage (Table 2). Additionally, hatching of 100% of these larvae was delayed by 24–48 hours. Thus, co-expression of both APP and Tip60^E431Q^ resulted in a much more severe developmental phenotype as it induced lethality approximately 3 days earlier in development than when compared to either APP or dTip60^E431Q^ expressed alone. The genetic enhancement of lethal effects observed in the double mutants compared to when either APP or dTip60^E431Q^ is expressed alone is indicative of a synergistic interaction between Tip60 and APP.

**Table 2:** Developmental stage at which expression of the different transgenes induces lethality.

Transgenic fly lines^a^	Developmental Stage of Lethality^b^	
	Ubiquitous expression^c^	Pan-neuronal expression^d^
Wild type (*w^1118^*)	Not lethal	Not lethal
APP	Pupae/Adult	Pupae/Adult
APP dCT	Not lethal	Not lethal
dTip60 ^E431Q^ A	Late 3^rd^ instar	Late 3^rd^ instar*
APP; dTip60 ^E431Q^ A	Early 2^nd^ instar (hatching delayed by 24–48 hrs)	Early 2^nd^ instar*
APP dCT; dTip60 ^E431Q^ A	Late 3^rd^ instar	Late 3^rd^ instar*
dTip60 ^E431Q^ B	Late 3^rd^ instar	Late 3^rd^ instar
APP; dTip60 ^E431Q^ B	Early 2^nd^ instar (hatching delayed by 24–48 hrs)	Early 2^nd^ instar
APP dCT; dTip60 ^E431Q^ B	Late 3^rd^ instar	Late 3^rd^ instar
dTip60^WT^ lines A, B, C	Partially lethal	Partially lethal
APP; dTip60^WT^ lines A, B, C	Partially lethal	Partially lethal
APP dCT; dTip60^WT^ lines A, B, C	Partially lethal	Partially lethal

a Ten female virgin flies homozygous for the indicated transgene or control *w^1118^* were crossed to seven males homozygous for the Gal4 driver. All crosses were carried out in triplicate at 25°C.

b The crosses were monitored daily and the developmental stage at which lethality occurred was scored.

C The 337-Gal4 was used to drive ubiquitous expression of transgenes.

d The 179-Gal4 driver located on the X-chromosome was used to drive pan-neuronal expression of transgenes. * Neuronal expression of low expressing independent fly line dTip60 HAT mutant (dTip60^E431Q^ A) alone or in conjunction with APP/APP dCT induced lethality in a fraction of the respective F1 progeny at the indicated developmental stage while the remainder of F1 progeny did not exhibit any lethal effect.

To determine whether the genetic enhancement we observed between APP and dTip60 was dependent upon the C-terminal domain of APP that is required for interaction with dTip60, we co-expressed the dTip60^E431Q^ transgene with APP dCT, a version of APP lacking the C-terminal domain. Ubiquitous expression of APP dCT alone with the 337-GAL4 driver at 25°C did not cause any observable developmental phenotype although there was a non-significant decrease in the number of F1 progeny that eclosed (Table 2, Figure 3). However, unlike the APP eclosed flies that survived only 2–5 days (Table 2), the eclosed APP dCT adult progeny in this case did not exhibit any early lethality. This finding indicates that the decrease in viability in response to APP overexpression is dependent upon the C-terminus domain of APP. Moreover, co-expression of dTip60^E431Q^ with APP dCT using both APP dCT; dTip60^E431Q^ line A and APP dCT; dTip60^E431Q^ line B resulted in a phenotype identical to that of dTip60^E431Q^ alone (Table 2). These results indicate that the synergistic interaction between dTip60^E431Q^ and APP is dependent upon the Tip60 interacting C-terminal domain of APP.

We also examined the effect of overexpressing varying levels of wild type dTip60 using dTip60 ^WT^ lines A, B and C using the ubiquitous 337-Gal4 driver. As shown in Table 2, ubiquitous expression of each of these transgenes did not affect development *per se* but the number of F1 progeny that eclosed in each case was significantly less than the wild type control (Figure 3). Although these dTip60 ^WT^ lines express varying levels of the wild type dTip60, there was no significant difference in the number of surviving F1 progeny between dTip60 ^WT^ lines A, B and C indicating that the observed effect is not dose dependent. In contrast, co-expression of dTip60^WT^ with APP using lines APP; dTip60^WT^ A, B and C rescued the APP induced loss of viability in a dose dependent fashion, as indicated by the increase in the number of surviving F1 progeny in the double mutants compared to flies expressing APP alone (Figure 3). However, the number of F1 progeny was still less than the wild type control in all three cases indicating only a partial rescue of the APP induced lethality. Notably, with APP; dTip60^WT^ line C that co-expresses APP with the highest level of wild type Tip60, the number of F1 progeny that eclosed was significantly more than that observed in the respective single mutant dTip60^WT^ lines (Figure 3). Thus, co-expression of APP with additional levels of Tip60 not only counteracts the lethal effects induced by APP but also alleviates the effect that overexpression of Tip60 has on viability. Lack of similar effects in the APP dCT; dTip60^WT^ C flies (Figure 3) suggest that the observed rescue phenotype was mediated through interaction of Tip60 with the APP C-terminal domain. Together, our findings indicate that while loss of Tip60 HAT activity enhances the APP induced lethal effects, additional levels of Tip60 suppress such lethal effects, further supporting a synergistic interaction between Tip60 and APP.

APP and Tip60 are each neuronally expressed and are both required for nervous system function [4], [27]. Thus, the phenotypic enhancement we observed between APP and Tip60 during general development prompted us to ask whether this interaction was also specific for nervous system development and function. To investigate whether Tip60 and APP genetically interact in the nervous system, we carried out the same crosses as above, this time using the pan-neuronal 179 y- GAL4 driver line which induces robust pan- neuronal GAL4 expression at 25°C (Table 2). Again, we observed the same pattern of lethality as for general development for the stronger fly line APP; Tip60^E431Q^ B in that lethality caused by APP overexpression was enhanced by reduction of Tip60 HAT activity, supporting the specificity of the Tip60 and APP genetic interaction (Table 2) in nervous system development. As before, this nervous system specific interaction was dependent upon the Tip60 interacting C-terminal domain of APP (Table 3). In contrast, when Tip60^E431Q^ A was expressed in the nervous system in combination with APP or APP dCT, it resulted in partial lethality wherein only a fraction of the F1 progeny in each of these cases died as second and third instars, respectively similar to that seen in APP; Tip60^E431Q^ B and APP dCT; Tip60^E431Q^ B flies. However, the majority of F1 progeny did not have any lethal developmental effect (Table 2). This milder effect observed with Tip60^E431Q^ A expressing flies is likely due to the low level of dTip60 HAT mutant that is expressed in these flies. Similar to the effects we observed with ubiquitous expression, pan neuronal expression of dTip60^WT^ with APP suppressed the APP induced lethality in a dose dependent fashion (Figure 3). Furthermore, with APP; dTip60^WT^ line C, the number of F1 progeny that eclosed were significantly more than that observed in the respective single mutant dTip60^WT^ lines (Figure 3). Taken together, our results demonstrate that Tip60 and APP functionally interact to mediate both general and nervous system specific development and that this interaction is dependent upon the Tip60 interacting C-terminal domain of APP. These data further support an epigenetic based role for Tip60 HAT activity in mediating APP induced developmental effects.

**Table 3:** Apoptosis pathways significantly misregulated in response to dTip60 HAT loss.

Apoptosis related pathway	Number of genes
Alzheimer disease – presenilin pathway	11
Angiogenesis	28
Apoptosis signaling pathway	19
ATP synthesis	3
Denovo purine biosynthesis	15
Denovo pyrimidine deoxyribonucleotide biosynthesis	7
Denovo pyrimidine ribonucleotide biosynthesis	6
EGF receptor signaling pathway	26
FAS signaling pathway	8
FGF signaling pathway	28
Huntington disease	37
Integrin signaling pathway	32
Notch signaling pathway	4
Oxidative stress response	11
P53 pathway	33
Parkinson disease	15
Wnt signaling pathway	46

### Tip60 HAT activity is required for the transcriptional regulation of genes linked to a variety of distinct apoptotic pathways

**Figure 4:**
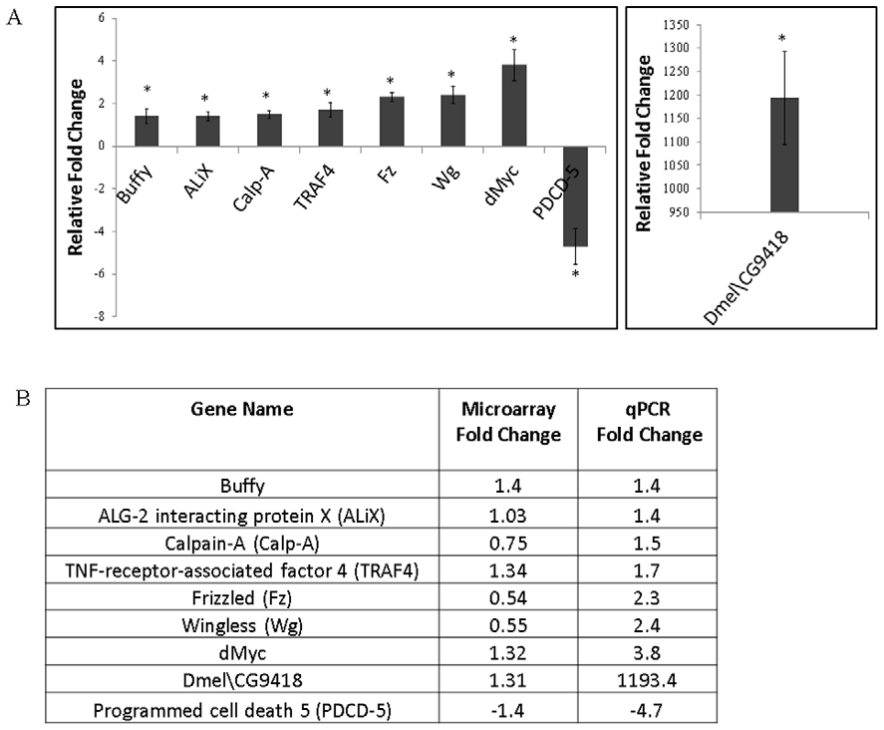
Quantitative RT-PCR validation of selected apoptosis related genes identified by microarray analysis. (**A**) Histogram showing relative fold change in expression level of apoptosis related target genes in flies expressing dTip60^E431Q^ A. Staged second instar larvae were used for cDNA preparation. RT-PCR reactions were carried out in triplicate and the fold change was calculated using the 2−ΔΔCT method using RP49 as control. (**B**) List of selected apoptosis related target genes identified by microarray analysis and validated in the dTip60^E431Q^ A line using quantitative RT-PCR.

The above findings indicating a functional interaction between APP and Tip60 in mediating general and nervous system specific lethality prompted us to ask whether a potential mechanistic basis for this lethal phenotype was *via* induction of an apoptotic response in these flies. We recently reported a microarray analysis comparing global changes in gene expression in response to ubiquitous induction of Tip60^E431Q^ in the fly [4]. While this study reported misregulation of genes linked to diverse neuronal functions, the identity of genes that function in specific neuronal processes was not explored. To address this and to examine the causative mechanism that mediates the Tip60/APP induced lethal phenotype, we wanted to further analyze our previously published microarray gene expression data with specific focus on genes that are known to function in apoptosis related pathways. Towards this end, we performed pathway analysis by first identifying canonical apoptotic pathways and their respective genes from online databases like Gene Ontology and the PANTHER classification system. The dTip60^E431Q^ microarray data set was then examined to see if genes linked to such apoptotic pathways were misregulated in response to loss of Tip60's HAT activity. Our analysis identified 53 such unique genes that are involved in 17 different apoptotic pathways to be misregulated in the dTip60^E431Q^ data set (Table 3). Intriguingly, the identified pathways included those that are associated with Alzheimer's, Parkinson's and Huntington's diseases, all neurodegenerative disorders in which massive neuronal death due to apoptosis is a common characteristic. Importantly, the p53 mediated pathway and Wnt signaling pathway were among the most highly represented pathways, consistent with previous reports implicating Tip60 in a p53 mediated apoptotic response. To validate our microarray results, we carried out quantitative RT-PCR analysis of nine genes that encoded protein products with known functions involved in inducing an apoptotic response (Figure 4A and 4B) and were representative of a particular pathway (Table 3). Of the genes that were upregulated in response to Tip60 HAT loss was Calpain, a calcium dependent enzyme that mediates proteolytic cleavage of proteins like APP and tau. Abnormal activation of Calpain has also been reported to initiate degradation of proteins essential for neuronal survival [13]. Among the other confirmed targets that were upregulated were genes with established roles in the induction of the p53 mediated apoptotic pathway such as TRAF4 and CG9418 (High mobility group protein 1/2). The wingless protein (wg) and Frizzled (Fz), a transmembrane protein that functions as Wg receptor were two confirmed upregulated targets critical in the Wnt signaling pathway involved in regulating apoptosis. Also upregulated in the microarray data was ALiX (apoptosis linked gene 2 interacting X), a calcium dependent ubiquitously expressed protein involved in neuronal cell death. Consistent with this finding, upregulation of endogenous ALiX has also been reported to correlate with cell death *in vivo* [27], [28]. Myc proteins are essential regulators of cellular growth and proliferation during normal development. Recently, the ability of overexpressed Myc to induce cell-autonomous apoptosis has been shown to be evolutionarily conserved in *Drosophila* Myc [29]. Interestingly, we too found Myc to be upregulated in response to loss of Tip60 HAT activity. Our identification of these target genes that are affected by loss of Tip60 HAT activity further support an as yet unidentified putative role for Tip60 in the respective cellular pathways in which such targets function. Among the genes downregulated in response to loss of Tip60 HAT activity was the apoptosis related protein, Programmed Cell Death 5 (PDCD5) that has also been reported to interact with Tip60 to mediate DNA damage induced apoptosis [30]. In summary, our identification of misregulated apoptosis related pathways and their respective genes in response to Tip60 HAT loss support a transcriptional regulatory role for Tip60 in multiple pathways linked to apoptotic control.

In order to examine if expression of these genes that are misregulated in dTip60^E431Q^ are also altered due to overexpression of wild type dTip60, we performed qPCR analysis of the above mentioned nine genes in dTip60^WT^ second instar larvae, as this was the developmental stage used for dTip60^E431Q^ microarray analysis (Table 4). While loss of Tip60 HAT activity induced expression of genes like Frizzled, Wingless and dMyc, Tip60 overexpression had the converse effect resulting in marked downregulation of these genes. Significant differential regulation was also observed between dTip60^E431Q^ and dTip60^WT^ flies for PDCD5 expression. Similar to that observed in the Tip60 HAT mutants, expression of genes like Buffy, ALiX, CalpA, TRAF4 was also induced under Tip60 overexpressing conditions (Table 4).

**Table 4:** Gene expression changes of dTip60^E431Q^ misregulated target genes in the different transgenic lines.

Gene Name^a^	Transgenic Fly Line (Relative Fold Change)^b^			
	dTip60^E431Q^	dTip60^WT^	APP; dTip60^E431Q^	APP; dTip60^WT^
Buffy § ^, ^ 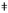	1.4	1.6	−1.5	3.5
ALiX	1.4	2.1	1.5	2.3
CalpA § ^, ^ 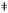	1.5	3.5	−2.1	−1.8
TRAF4 § ^, ^ 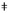	1.7	3.9	−1.5	−1.7
Frizzled §	2.3	−1.5	−2.1	−1.5
Wingless §	2.4	−1.7	−1.9	−1.5
dMyc §	3.8	−3.2	−2.5	−2.3
PDCD5 §	−4.7	2	1	1.8
Dmel\CG9418 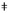	1193.4	14.8139954	824.094897	2472.348951

a Quantitative RT-PCR analysis was performed for the indicated target genes.

b Staged second instar larvae ubiquitously expressing the indicated transgene(s) were used for cDNA preparation. Quantitative RT-PCR reactions were carried out in triplicate and the relative fold change was calculated using the 2−ΔΔCT method using RP49 as control.

§ Genes that were differentially regulated between flies expressing the Tip60 HAT mutant dTip60^E431Q^ alone and in conjunction with APP.

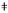
Gene that were differentially regulated between flies overexpressing dTip60^WT^ alone or together with APP.

Since Tip60 forms a transcriptionally active complex with the APP C-terminal domain, we also wished to examine how these gene expression changes are modified by APP in the dTip60^E431Q^ or dTip60^WT^ background. We therefore performed qPCR analysis of these nine genes in APP; dTip60^E431Q^ and APP; dTip60^WT^ double mutant lines to identify genes that are differentially regulated between these lines and their respective single mutants (Table 4). Notably, while Tip60 HAT loss in dTip60^E431Q^ fly lines induced expression of the genes Buffy, CalpA, TRAF4, Frizzled, Wingless, dMyc, co-expression of APP with dTip60^E431Q^ had a repressive effect on each of these genes. Similar differential regulation was observed with PDCD5 wherein the presence of APP with dTip60^E431Q^ relieved the repressive effect on PDCD5 that expression of dTip60^E431Q^ alone had. With respect to APP; dTip60^WT^ flies, CalpA, TRAF4 and Dmel\CG9418 each exhibited differential regulation in comparison to flies expressing dTip60^WT^ alone. While CalpA and TRAF4 were upregulated in dTip60^WT^ flies, they were downregulated in APP; dTip60^WT^ flies. Although Dmel\CG9418 was upregulated in dTip60^WT^ flies, its fold increase was much higher in APP; dTip60^WT^ flies. Finally, Buffy was significantly upregulated in the APP;dTip60^WT^ flies when compared to flies expressing dTip60^WT^ alone (Table 4). Taken together, these results indicate that Tip60 target gene expression profiles can be modified in the presence of APP.

### TIP60 and APP functionally interact to mediate apoptotic cell death in the *Drosophila* CNS

Our finding that Tip60 and APP genetically interact to specifically mediate nervous system development prompted us to ask what specific neuronal processes might be regulated by this interaction. Targeted overexpression of APP in the *Drosophila* nervous system was previously shown to induce neuronal apoptosis in the CNS at 29°C, [25], however whether this phenotype can be induced at normal physiological temperature as well as the mechanism underlying such apoptotic induction remain to be elucidated. Moreover, and in agreement with previous reports, here we show that Tip60 HAT activity controls apoptotic pathways *via* the transcriptional regulation of apoptosis linked genes. These findings prompted us to ask whether dTip60 and APP genetically interact to mediate apoptotic neuronal cell death in the *Drosophila* CNS.

**Figure 5:**
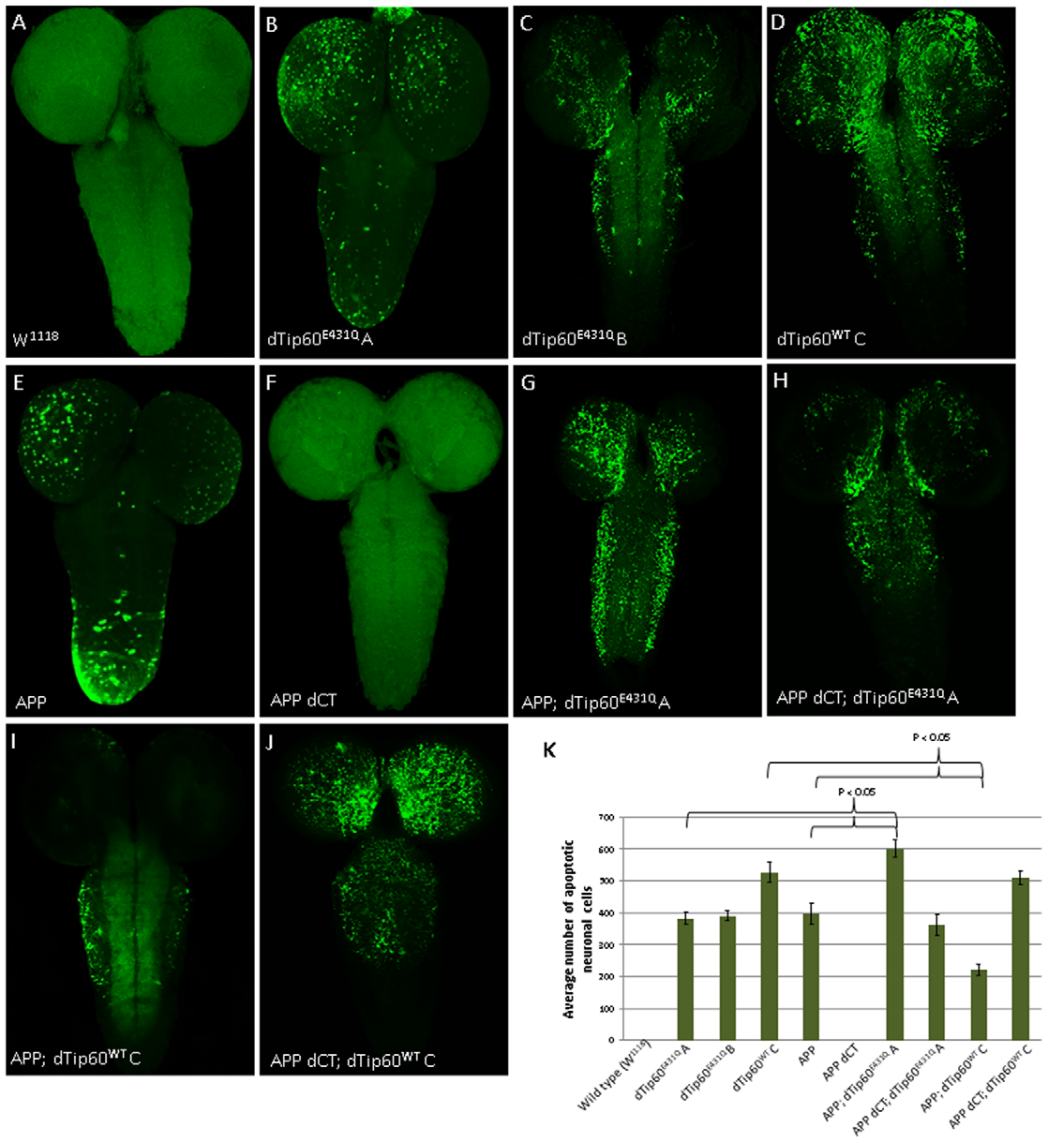
dTip60 mediates APP induced apoptotic neuronal cell death in the *Drosophila* central nervous system. Representative confocal images of neuronal apoptosis visualized by TUNEL staining of brains from staged third instar larvae expressing indicated transgenes driven by pan-neuronal driver 179-GAL4. The *w^1118^* larvae used as genetic background control showed no apoptosis (**A**). Pan neuronal expression of dTip60^E431Q^ induces apoptosis in a dose independent manner as evident from comparable levels of apoptosis seen in fly lines expressing low (**B**) or high (**C**) levels of dTip60^E431Q^. Overexpression of wild type dTip60 increased neuronal cell death due to apoptosis (**D**). The C-terminal domain of APP induces apoptosis as evident from TUNEL positive apoptotic cells in flies overexpressing APP (**E**) while no apoptosis was observed in flies expressing a truncated version of APP lacking the C-terminal domain (**F**). Co-expression of APP with low levels of dTip60^E431Q^ (dTip60^E431Q^ A) enhances the severity of apoptosis phenotype in a synergistic manner (**G**) that is dependent on the APP C-terminal domain. Co-expression of APP lacking C-terminus with dTip60^E431Q^ A exhibited apoptosis that was comparable to that seen when dTip60^E431Q^ A was expressed alone (**H**). Overexpression of wild type dTip60 in the APP overexpressing background partially rescued the apoptosis phenotype (**I**) but in the presence of APP lacking C-terminus exerted similar severity seen in flies overexpressing wild type dTip60 alone. Images shown represent projections of 1 um confocal slices. Apoptotic cells in the different genotypes were quantified by counting the number of TUNEL positive cells in the entire fly brain (**K**).

To first determine whether misregulation of dTip60 levels causes neuronal specific apoptosis, Tip60^E431Q^ and Tip60 ^WT^ fly lines were crossed to the 179 y-GAL4 pan-neuronal driver flies at 25°C. The *w^1118^* fly line crossed to 179 y-GAL4 served as a control. Third instar larval brains were dissected from the progeny of these crosses and tested for apoptosis using dUTP nick end labeling (TUNEL) staining. As seen in Figure 5B and 5C, moderate levels of apoptotic induction were observed in larval brains of transgenic lines expressing either dTip60^E431Q^ A or dTip60^E431Q^ B while higher levels of apoptotic death were found for flies expressing comparable levels of Tip60^WT^ (Figure 5 compare B, C and D; Figure 5K). These results indicated that appropriate regulation of Tip60 levels play a critical role in controlling the balance of neuronal apoptotic cell death in the larval brain and that overexpression of Tip60 may be more detrimental than Tip60 HAT loss in this process. TUNEL staining of third instar larval brains from APP and APP dCT flies crossed to 179 y-GAL4 at 25°C were also assessed to determine whether APP overexpression induces neuronal apoptosis at physiological temperature and whether APP induced cell death is dependent upon its C-terminal domain, respectively. As shown in Figure 5E, moderate levels of apoptotic death were observed for APP overexpression at 25°C while no apoptosis was detected for flies expressing equivalent levels of APP dCT (Figure 5F). Furthermore, the extent of apoptosis induced by APP overexpression was comparable to that observed in both dTip60^E431Q^ A and dTip60^E431Q^ B flies (Figure 5K). These results indicated that APP overexpression induces neuronal apoptosis at physiological temperature, and that this phenotype is dependent upon its C-terminal domain, consistent with previous findings [25].

Given that Tip60 and APP each separately induced neuronal apoptosis in the *Drosophila* CNS, and that APP induced cell death was dependent upon its Tip60 interacting C-terminal domain, we predicted that Tip60 and APP might functionally interact to induce apoptosis mediated neurodegeneration when misregulated. To test this possibility, we first performed TUNEL assays in larval brains co-expressing either Tip60^E431Q^ and APP or Tip60^E431Q^ and APP dCT under the control of the pan-neuronal 179 y-GAL4 driver. For these studies, we used our lower expressing APP; Tip60^E431Q^ line A and APP dCT; Tip60^E431Q^ line A fly lines (Figure 1A), as co-expression of higher expressing Tip60^E431Q^ line B and APP induced lethality at the second instar larvae stage which was too early to assess by TUNEL stain. Indeed, as shown in Figure 5G, co-expression of Tip60^E431Q^ and APP resulted in a marked induction of apoptosis that was more robust than either Tip60^E431Q^ or APP alone (Figure 5K), indicative of a synergistic interaction between Tip60 and APP in neuronal apoptotic induction. Importantly, and as we predicted, this interaction was dependent upon the C-terminus of APP that interacts with Tip60 (Cau and Sudhoff, 2001) as co-expression of Tip60^E431Q^ and APP dCT resulted in only a moderate level of neuronal apoptosis induction that was approximately equivalent to that observed for Tip60^E431Q^ alone (Figure 5H and 5K). To determine whether additional Tip60 levels would suppress the APP induced neuronal apoptotic phenotype as well as to confirm the specificity of the interaction, we performed TUNEL assays in larval brains co-expressing Tip60^WT^ with APP using APP; Tip60^WT^ line C. This line was selected because line Tip60^WT^ C expressed the highest levels of wild type dTip60 for all of our dTip60^WT^ lines (Figure 2A) and also displayed the highest level of rescue for APP induced lethality (Figure 3). Remarkably, we found that additional levels of Tip60 partially rescued APP induced apoptotic cell death as evidenced by a visible reduction of the presence of TUNEL-positive cells in these brains when compared to APP alone (Figure 5I, compare 5E and 5I; Figure 5K). Co-expression of dTip60^WT^ and APP also appeared to suppress neuronal apoptosis induced by Tip60 overexpression alone, as we observed less TUNEL-positive cells in brains co-expressing dTip60^WT^ and APP when compared with brains expressing equivalent levels of Tip60^WT^ alone (Figure 5, compare D and I). Interestingly, rescue of cell death appeared more prominent in the proximal central brain of APP; dTip60^WT^ flies, as we consistently observed virtually no apoptotic cell death in this area (Figure 5I), where vital structures like the *Drosophila* learning and memory center mushroom body are located. Importantly, and as we predicted, partial rescue of APP induced neuronal apoptosis by Tip60 was dependent upon the Tip60 interacting C-terminus of APP, as brains co-expressing both Tip60^WT^ and APP dCT showed no rescue as shown by the equivalent number of TUNEL positive cells in these brains compared to those expressing Tip60^WT^ alone (Figure 5J and 5K). Taken together, our results demonstrate that Tip60 and APP functionally interact to regulate neuronal apoptotic cell death in the *Drosophila* CNS and that this interaction is dependent upon the C-terminus of APP.

## Discussion

In this study, we have generated a unique transgenic *Drosophila* model system suitable for investigating a functional link between Tip60 HAT activity and APP in neuronal development, *in vivo*. We demonstrate that Tip60 and APP functionally interact in both general and nervous system development in *Drosophila*, *in vivo* and that this interaction specifically mediates apoptotic neuronal cell death in the CNS, a process that when misregulated is linked to AD pathology [31]. Remarkably, Tip60 appears to display a neuroprotective function in that Tip60 overexpression can rescue both loss of viability and neuronal apoptosis induction in a *Drosophila* AD model. While a number of *in vitro* studies supporting the transcription regulatory role of the Tip60/AICD complex in gene control have been reported, our work is the first to demonstrate a functional interaction between Tip60 HAT activity and APP in nervous system development *in vivo*.

Here we show that misexpression of Tip60 induces neuronal apoptotic cell death in the *Drosophila* CNS, and that this process is mediated *via* a functional interaction between Tip60 and the APP C-terminal domain. Since disruption of Tip60 HAT activity induced neuronal cell death, we examined whether there was specific misregulation of apoptosis linked genes due to loss of Tip60 HAT activity. Pathway analysis of our previously reported microarray data set of genome wide changes in gene expression induced in the fly in response to Tip60 HAT loss [4] revealed genes functioning in 17 different apoptotic pathways to be enriched, many of which were associated with the p53 apoptotic pathway. Our findings are consistent with previous studies demonstrating a role for Tip60 as a p53 co-activator in p53 mediated apoptotic pathways [32]. Recent studies have found Tip60 to be required for activation of proapoptotic genes through acetylation of p53 DNA binding domain [16], [32]. TRAF4, one such p53 regulated pro-apoptotic gene [33] that responds to cellular stress was one of the genes that we found to be significantly upregulated in response to Tip60 HAT loss. The Myc family of transcription factors presents another instance of proteins involved in inducing apoptosis that are directly acetylated and stabilized by Tip60 [29] and accordingly, *Drosophila* dMyc was found to be significantly upregulated in response to Tip60 HAT loss. Thus it is possible that the pro-apoptotic genes enriched in our dataset may represent both direct targets regulated by Tip60 epigenetic function as well as indirect targets of apoptosis regulators such as p53 that are controlled *via* their acetylation by Tip60. Misregulation of these pro-apoptotic genes in response to disruption of Tip60 HAT activity is also consistent with our observation that nervous system specific expression of dTip60^E431Q^ induces apoptotic cell death in the CNS of dTip60^E431Q^ larvae. This finding is in contrast to previous studies wherein cells expressing mutated Tip60 lacking HAT activity were reported to be resistant to apoptosis. However, these studies examined a role for Tip60 in DNA damage repair following cellular stress using the H4 neuroglioma cells *in vitro*. WhileTip60 HAT activity is vital for DNA repair competency as well as for the ability to signal the presence of damaged DNA to the apoptotic machinery [34], how Tip60 HAT activity regulates differential gene expression profiles to prevent unwanted neuronal cell death during organismal development remains unclear. A number of mammalian studies have indicated that Tip60 can function not only as a coactivator, but also as a corepressor [35], [36] and as such, Tip60 has been shown to repress a vast array of developmental genes during ESC differentiation to maintain ESC identity [37]. Consistent with these findings, the majority of pro-apoptotic genes we identified that were misregulated in response to disruption of Tip60 HAT activity were upregulated, highlighting the crucial role Tip60 HAT activity plays in repression of apoptotic genes during neurogenesis that when misregulated, likely contribute to dTip60^E431Q^ induced apoptosis.

Interestingly, we find that overexpression of wild type Tip60 in the nervous system also induced apoptosis in the CNS. Furthermore, overexpressing Tip60 was found to induce expression of pro-apoptotic genes such as ALiX and CalpA while downregulating others like Wingless, Frizzled and dMyc that have multiple essential functions during *Drosophila* development. These bidirectional gene expression changes suggest that increasing Tip60 mediated acetylation can also lead to complex changes in the chromatin landscape resulting in inappropriate activation and/or repression of apoptosis competent genes as well as those crucial for development. Accumulating evidence shows that hyperacetylation can be fatal to neurons. Under normal conditions, increasing hyperacetylation by treating neurons with a general HDAC inhibitor like trichostatin A has been found to induce neuronal apoptosis [38], [39]. Similarly, increasing acetylation levels by overexpressing the HAT CBP in resting neurons has been reported to enhance chromatin condensation and neuronal death [15]. In order to maintain cellular homeostasis, HAT/HDAC equilibrium and therefore histone acetylation is strictly regulated as it is essential to maintain the functional status of neurons [40]. Based on these findings, we can speculate that overexpression of Tip60 disrupts the acetylation balance, thus skewing the neuronal survival pathway towards apoptosis and ultimately cell death. In support of this concept, altered levels of global histone acetylation have been observed in many *in vivo* models of neurodegenerative diseases [41], [42].

Another striking feature of our apoptotic microarray gene enrichment search was our identification of apoptosis linked pathways associated with neurodegenerative diseases like Parkinson's, Huntington's and Alzheimer's disease. These diseases are also characterized by neuronal cell death that increases over time and underlies an array of symptoms that depend on the function of the lost neuronal population [40]. It has been proposed that in AD, in addition to the deposition of toxic β-amyloid plaques in the brain, neurodegeneration may also be caused *via* γ-secretase cleavage of APP that generates AICD carboxy terminal fragments that are toxic to neurons [18]. Accordingly, ectopic expression of AICD in rat pheocytoma cells and cortical neurons [43] and H4 neuroglioma cells [18] has been shown to induce apoptosis upon nuclear translocation. Consistent with these reports, we too observe induction of apoptosis when APP is expressed in the nervous system of *Drosophila in vivo* at physiological temperatures and that this phenotype is dependent upon the C-terminal domain of APP. Interestingly, APP C-terminal domain induced apoptosis has previously been reported to be mediated *via* Tip60 HAT activity *in vitro*, such that induction of apoptosis in neuroglioma cells transfected with APP C-terminal domain is enhanced by co-transfection of wild type Tip60 and decreased by a dominant negative version of Tip60 lacking HAT activity [18]. In contrast, here we demonstrate that nervous system specific co-expression of APP and HAT defective mutant Tip60 increases apoptosis while overexpression of wild-type Tip60 with APP counteracts this effect and that these phenotypes are dependent upon the Tip60 interacting C-terminus of APP. Such differences may be accounted for by the fact that we are carrying out our studies in a developmental model system, *in vivo*. However, the effects we show on neuronal apoptosis are also consistent with the effects we observed in the viability assay wherein lethality caused by neuronal overexpression of APP was enhanced by reduction of Tip60 HAT activity and suppressed by additional Tip60 levels. Importantly, this finding, in conjunction with our previously published reports supporting a causative role for Tip60 in the control of synaptic plasticity [5] and the transcriptional regulation of genes enriched for neuronal function [4], support the concept that misregulation of Tip60 HAT activity can lead to aberrant gene expression within the nervous system that contributes to the AD associated neurodegenerative process.

Tip60 has been implicated in AD *via* its transcriptional complex formation with AICD [6], [9]. Thus, we carried out experiments to determine whether the expression of specific genes that are misregulated by dTip60^E431Q^ or dTip60^WT^ are modified by the presence of APP. Intriguingly, we found a number of these genes to be differentially regulated under APP expressing conditions. Two such genes, Wingless and Frizzled, which are upregulated in dTip60^E431Q^ flies and repressed in dTip60^WT^ flies are particularly interesting. Wingless, the *Drosophila* segment polarity gene and its membrane receptor Frizzled are known to be required for specification and formation of various neurons in the CNS [44] and belong to the Wnt signaling pathway. In addition to Wingless and Frizzled being important for the disease process, they are also crucial for normal growth and development. Intriguingly, we find that co-expressing APP with either the Tip60 HAT mutant or in the Tip60 overexpressing background has a repressive effect on these essential genes. Recent evidence supports a neuroprotective role for the Wnt signaling pathway [45], [46] and a sustained loss of Wnt signaling function is thought to be involved in aβ induced neurodegeneration [47]. *Drosophila* Myc is a regulator of rRNA synthesis and is necessary for ribosome biogenesis during larval development [48] and is another instance of a vital gene that exhibited reduced expression under APP expressing conditions. Thus misregulation of such developmentally required genes in conjunction with the other pro-apoptotic genes in our data set likely contributed to the observed enhanced apoptotic cell death in the CNS of APP;dTip60^E431Q^ larvae. In contrast, we find the *Drosophila* homolog of Bcl-2 protein, Buffy to be repressed in the APP; dTip60^E431Q^ flies that displayed an increase in apoptosis. Consistent with our findings, recent studies have reported that Buffy has anti-apoptotic functions *in vivo* [49] and intriguingly, we find its expression to be significantly induced in the APP; dTip60^WT^ flies that also exhibited a marked reduction in apoptosis induced cell death when compared to flies expressing dTip60^WT^ alone. These findings suggest that induction of such pro-survival factors could mediate the dTip60 induced rescue of APP mediated defects that we observe in these flies.

We observe differential regulation of the microarray targets between flies that express dTip60^E431Q^ alone and in conjunction with APP, in that the majority of genes we tested are repressed in the APP;dTip60^E431Q^ double mutants and activated in dTip60^E431Q^ flies. These results indicate that the presence of APP can modulate the transcriptional regulatory potential of Tip60. The APP intracellular domain was recently shown to lower the sensitivity of neuronal cells to toxic stimuli and transcriptionally activate genes involved in signaling pathways that are not active under basal conditions [50]. APP could mediate such effects either by sequestering Tip60 away from its typical target promoters or by displacing another factor in the complex that is also required for regulating transcription. Additionally, Tip60 has been shown to function as a negative regulator of gene expression. In fact, overexpression of Tip60 but not its HAT deficient mutant has been reported to function as co-repressor for gene repression mediated by transcription factors like STAT3 and FOX3, an effect that is mediated through association with specific histone deacetylases [51], [52]. This could partly account for the repressive effects that we observe due to overexpression of wild type Tip60 either alone or in conjunction with APP. Tip60 can also function as a co-activator of gene transcription *via* displacement of co-repressors on the promoters of specific genes. For instance, in a study by Baek *et al* [10], it was reported that following IL-1 stimulation, recruitment of a wild type Tip60 containing co-activator complex leads to activation of p50 target genes like KAI1/CD82 through displacement of a specific NCoR co-repressor complex. Intriguingly, the Tip60-FE65-AICD containing complex was shown to similarly displace the NCoR complex and derepress such targets, suggesting a potential transcription activation strategy that underlies the gene expression changes we observe under APP overexpressing conditions. Since loss of Tip60 HAT activity enhances APP induced lethal effects in the nervous system and overexpression of wild type Tip60 diminishes these defects, we hypothesize that the Tip60-AICD containing complex may mediate these rescue effects either *via* regulation of a subset of gene targets different from those targeted by either APP or Tip60 alone or by differentially regulating the same gene pool such as that seen in the case of the anti-apoptotic gene Buffy. Thus, although the repertoire of genes that we tested include both mediators as well as inhibitors of apoptosis, taken together our data support a model by which Tip60 HAT activity plays a neuroprotective role in disease progression by complexing with the AICD region of APP to epigenetically regulate transcription of genes essential for tipping the cell fate control balance from apoptotic cell death towards cell survival under neurodegenerative conditions such as excess APP. We therefore propose a neuroprotective role for Tip60 in AD linked induction of apoptotic cell death. Future investigation into the mechanism by which Tip60 regulates these processes may provide insight into the utility of specific HAT activators as therapeutic strategies for neurodegenerative disorders.
